# Ivabradine increases the high frequency gain ratio in the vagal heart rate transfer function via an interaction with muscarinic potassium channels

**DOI:** 10.14814/phy2.15134

**Published:** 2021-12-09

**Authors:** Toru Kawada, Hiromi Yamamoto, Tadayoshi Miyamoto, Yohsuke Hayama, Meihua Li, Can Zheng, Kazunori Uemura, Masaru Sugimachi, Keita Saku

**Affiliations:** ^1^ Department of Cardiovascular Dynamics National Cerebral and Cardiovascular Center Osaka Japan; ^2^ Department of Cardiology Kurashiki Central Hospital Ohara HealthCare Foundation Okayama Japan; ^3^ Division of Clinical Research Kurashiki Clinical Research Institute Ohara HealthCare Foundation Okayama Japan; ^4^ Department of Sport and Health Sciences Faculty of Sport and Health Science Osaka Sangyo University Osaka Japan

**Keywords:** ivabradine, transfer function, vagal nerve stimulation, white noise

## Abstract

Muscarinic potassium channels (*I*_K,ACh_) are thought to contribute to the high frequency (HF) dynamic heart rate (HR) response to vagal nerve stimulation (VNS) because they act faster than the pathway mediated by hyperpolarization‐activated cyclic nucleotide‐gated (HCN) channels. However, the interactions between the two pathways have not yet been fully elucidated. We previously demonstrated that HCN channel blockade by ivabradine (IVA) increased the HF gain ratio of the transfer function from VNS to HR. To test the hypothesis that IVA increases the HF gain ratio via an interaction with *I*_K,ACh_, we examined the dynamic HR response to VNS under conditions of control (CNT), *I*_K,ACh_ blockade by tertiapin‐Q (TQ, 50 nM/kg), and TQ plus IVA (2 mg/kg) (TQ + IVA) in anesthetized rats (*n* = 8). In each condition, the right vagal nerve was stimulated for 10 min with binary white noise signals between 0–10, 0–20, and 0–40 Hz. On multiple regression analysis, the HF gain ratio positively correlated with the VNS rate with a coefficient of 1.691 ± 0.151 (×0.01) (*p* < 0.001). TQ had a negative effect on the HF gain ratio with a coefficient of −1.170 ± 0.214 (×0.01) (*p* < 0.001). IVA did not significantly increase the HF gain ratio in the presence of TQ. The HF gain ratio remained low under the TQ + IVA condition compared to controls. These results affirm that the IVA‐induced increase in the HF gain ratio is dependent on the untethering of the hyperpolarizing effect of *I*_K,ACh_.

## Graphical abstract

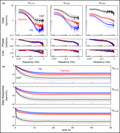

**NEW & NOTEWORTHY**

The interactions between two major mechanisms of vagal heart rate (HR) control, namely, the muscarinic potassium channel (*I*_K,ACh_) pathway and the hyperpolarization‐activated cyclic nucleotide‐gated (HCN) channel pathway, need to be fully elucidated. Blockade of HCN channels by ivabradine (IVA) reduces HR and increases the high frequency (HF) gain ratio of the transfer function from vagal nerve stimulation to HR in our previous studies. Under *I*_K,ACh_ blockade, IVA did not increase the HF gain ratio compared to controls in the present study. The IVA‐induced increase in the HF gain ratio is dependent on the untethering of the hyperpolarizing effect of *I*_K,ACh_.

## 1 INTRODUCTION

Two main pathways mediate the vagal control of heart rate (HR) in the sinoatrial nodal cells (Accili et al., 1998). One pathway is related to the current through the muscarinic potassium channels (*I*_K,ACh_), and the other pathway modulates the funny current (*I*_f_) through the hyperpolarization‐activated cyclic nucleotide‐gated (HCN) channels. The former is directly activated by βγ‐subunits of an inhibitory G protein (Nair et al., 1995), whereas the latter is indirectly regulated via changes in the intracellular concentration of cyclic adenosine monophosphate (cAMP). Sympathetic nerve stimulation increases the production of cAMP through a stimulatory G protein, and then, cAMP shifts the activation curve of *I*_f_ toward a more positive potential (DiFrancesco & Borer, 2007; DiFrancesco & Tortora, 1991). This shift increases the rate of slow diastolic depolarization and shortens the cycle length. Meanwhile, vagal nerve stimulation (VNS) antagonizes the production of cAMP through an inhibitory G protein and prolongs the cycle length. Compared to the cAMP‐mediated *I*_f_ pathway, the *I*_K,ACh_ pathway participates in a fast HR response because it does not involve cytoplasmic components. Despite the fundamental understanding of the two pathways, interactions between the two pathways are not yet fully elucidated in terms of dynamic vagal control of HR.

As HR changes dynamically in response to changes in autonomic nervous activities during daily activities, drugs affecting HR need to be evaluated in terms of their effect on the dynamic control of HR. Ivabradine (IVA) is a selective bradycardic agent that inhibits the HCN channels (isoform 4) in the sinoatrial nodal cells (DiFrancesco & Borer, 2007). In our previous study, IVA augmented the dynamic gain of the transfer function from VNS to HR during moderate‐intensity VNS (Kawada, Yamamoto, et al., 2019). This augmentation partly depended on sympathetic background tone because β‐blockade prevented the augmentation of the dynamic gain in the low frequency (LF, approximately 0.01–0.05 Hz) range (Kawada et al., 2021). On the other hand, β‐blockade did not prevent the augmentation of the dynamic gain in the high frequency (HF, approximately 0.5–1 Hz) range. When the ratio of the HF gain to the LF gain was calculated, IVA increased the HF gain ratio regardless of β‐blockade and differences in VNS intensity.

We speculated that interactions between the *I*_f_ and *I*_K,ACh_ pathways could explain the frequency‐dependent effect of IVA on the transfer function from VNS to HR. Because HCN channels are activated by hyperpolarization, they inherently counteract the hyperpolarizing effect of *I*_K,ACh_. The inhibition of HCN channels by IVA may untether the hyperpolarizing effect of *I*_K,ACh_ and may make the maximum diastolic potential (MDP) more negative during VNS, thus augmenting its bradycardic effect. Because the *I*_K,ACh_ pathway is faster than the cAMP‐mediated *I*_f_ pathway in regulating HR, this augmentation was prominent in the HF range. If this speculation is correct, the IVA‐induced increase in the HF gain ratio will disappear under *I*_K,ACh_ blockade. Hence, the present study aimed to test the hypothesis that IVA does not increase the HF gain ratio under *I*_K,ACh_ blockade. We estimated the transfer function from VNS to HR under conditions of control (CNT), *I*_K,ACh_ blockade by tertiapin‐Q (TQ), and TQ plus IVA (TQ+IVA) in anesthetized rats.

## 2 MATERIALS AND METHODS

This study conforms to the Guiding Principles for the Care and Use of Animals in the Field of Physiological Sciences, which has been approved by the Physiological Society of Japan. The experimental protocols were reviewed and approved (#21009) by the Animal Subjects Committee at the National Cerebral and Cardiovascular Center.

### 2.1 Surgical preparation

Male Wistar–Kyoto rats weighing 328–398 g (*n* = 8) were anesthetized by an intraperitoneal injection (2 ml/kg) of a mixture of urethane (250 mg/ml) and α‐chloralose (40 mg/ml). A maintenance dose (18‐fold diluted with physiological saline, 2–3 ml kg^−1^ h^−1^) was administered through the right femoral vein. Another venous line was prepared through the left femoral vein for drug administrations (TQ and TQ + IVA). Arterial pressure (AP) was measured from the right femoral artery. HR was determined from a body surface electrocardiogram. The rats were mechanically ventilated with room air supplemented with oxygen. The body temperature of the rat was maintained between 37°C and 38 °C using a heating pad and a lamp.

The bilateral vagal and aortic depressor nerves were sectioned at the neck. The bilateral carotid sinus baroreceptor regions were isolated from the systemic circulation (Sato et al., 1999; Shoukas et al., 1991), and the carotid sinus pressure was maintained at a prevailing AP through a servo‐controlled piston pump system. A pair of stainless‐steel wire electrodes (AS633, Cooner Wire) was attached to the sectioned right vagal nerve for unilateral efferent VNS. The nerve and electrodes were fixed and insulated with silicone glue (Kwik‐Sil, World Precision Instruments).

### 2.2 Protocols

After the surgical preparation was completed, 5‐min constant VNS (5 V, 0.1‐ms pulse width, the cathode electrode on the cardiac side) was applied several times with 5‐min intervals to establish stable stimulatory conditions. Thereafter, three VNS trials under different stimulation rates (10, 20, and 40 Hz) were performed under the CNT condition. In each VNS trial, stimulation was turned on and off every 500 ms according to a binary white noise sequence for 10 min. The three VNS trials were denoted as V_0–10_, V_0–20_, and V_0–40_. A 5‐min interval with no stimulation was allowed between VNS trials. Next, *I*_K,ACh_ was blocked by an intravenous administration of TQ (50 nM/kg, MedChemExpress). After a settling period of 10 min, the three VNS trials were repeated. Finally, a supplemental dose of TQ (50 nM/kg) and IVA (2 mg/kg, ivabradine hydrochloride, Tokyo Chemical Industry) was administered. After approximately 15 min, when the IVA‐induced HR reduction reached a steady state, the three VNS trials were repeated. The order of the three VNS trials was identical within the same rat but randomized for each rat.

TQ is a stable derivative of tertiapin (Kanjhan et al., 2005). The dose of TQ was determined based on our previous study in rabbits (Mizuno et al., 2007), in which intravenous tertiapin at 30 nM/kg significantly inhibited the dynamic HR response to VNS. Considering the prolonged protocol with three VNS trials under the TQ and TQ+IVA conditions, the dose was increased to 50 nM/kg in the present study. After completing the TQ +IVA condition, we confirmed that the addition of TQ at 50 nM/kg did not further change the HR response to VNS in two out of the eight rats. Hence, we think the dose of TQ might have been sufficient to block *I*_K,ACh_ in rats in vivo.

### 2.3 Data analysis

The VNS command, HR, and AP were recorded at a sampling rate of 1000 Hz. The transfer function from VNS to HR was estimated as follows. The data were processed starting at 2 min after the initiation of VNS. The VNS and HR data were resampled at 10 Hz and divided into eight half‐overlapping segments of 1024 points each. The linear trend was subtracted, and a Hanning window applied in each segment. The input power spectra [*S*_VNS·VNS_(*f*)], output power spectra [*S*_HR·HR_(*f*)], and cross spectra [*S*_HR·VNS_(*f*)] were calculated over the eight segments via Fourier transform. The transfer function from VNS to HR was estimated from the following equation (Equation 1) (Bendat & Piersol, 2010; Kawada, Mukkamala, et al., 2019):

(1) $$H\left(f\right)=\frac{S_{\mathrm{HR}\cdot \mathrm{VNS}}\left(f\right)}{S_{\mathrm{VNS}\cdot \mathrm{VNS}}\left(f\right)}.$$

The magnitude‐squared coherence function was also calculated from the following equation (Equation 2) (Bendat & Piersol, 2010; Kawada, Mukkamala, et al., 2019):

(2) $$\text{Coh}\left(\text{f}\right)\text{=}\frac{{\left|{\text{S}}_{\text{HR}\text{·}\text{VNS}}\left(\text{f}\right)\right|}^{\text{2}}}{{\text{S}}_{\text{VNS}\text{·}\text{VNS}}\left(\text{f}\right){\text{S}}_{\text{HR}\text{·}\text{HR}}\left(\text{f}\right)}\text{.}$$

The following mathematical model (Equation 3) was used to describe the estimated transfer function from VNS to HR according to previous studies (Kawada et al., 2013, 2021; Kawada, Yamamoto, et al., 2019):

(3) $$H_{\text{Model}}\left(f\right)=‐K\left(\frac{1‐R}{1+\frac{f}{f_{C}}j}+R\right)\exp\left(‐2\pi fLj\right),$$

where *j*, imaginary unit; *f*, frequency (Hz); *K*, steady‐state gain (bpm/Hz), referred to as “asymptotic LF gain”; *f*_C_, corner frequency (Hz); *R*, fraction of the HF gain to *K*, referred to as “HF gain ratio”; and *L*, dead time (s).

To promote intuitive understanding of the estimated transfer function, the HR step response was calculated from a time integral of the inverse Fourier transform of the transfer function. The step response was evaluated using the initial response at 1 s (*S*_1_), the steady‐state response averaged from 40 to 50 s (*S*_50_), and the ratio of *S*_1_ to *S*_50_.

In each VNS trial, the prestimulation HR and AP values was determined as the averaged data for 60 s before the onset of VNS. The mean HR and AP values during VNS were calculated using the data from 2 to 10 min of VNS. The delta HR was calculated as the difference of the mean HR during VNS from the prestimulation HR.

### 2.4 Statistical analysis

The data were presented as mean ± SE values. The effect of VNS and the differences among the CNT, TQ, and TQ +IVA conditions were analyzed via multiple regression analysis (Equation 4; Glantz & Slinker, 2001),

(4) $$\begin{array}{cc} p & =C+B_{\text{TQ}}\times D_{\text{TQ}}+B_{\text{IVA}}\times D_{\text{IVA}}\\ & \;+\left(B_{\text{VNS}}+B_{\text{VNS}\times \text{TQ}}\times D_{\text{TQ}}+B_{\text{VNS}\times \text{IVA}}\times D_{\text{IVA}}\right)\times N_{\text{VNS}}\\ & \;+B_{1}\times D_{1}+B_{2}\times D_{2}+\cdots +B_{7}\times D_{7} \end{array}$$

where *p*, tested parameter; *C*, constant describing the intercept of the multiple regression; *B*_TQ_ and *D*_TQ_, coefficients for the effect of TQ and the dummy variable, respectively, encoding the absence (*D*_TQ_ = 0) or presence (*D*_TQ_ = 1) of TQ; *B*_IVA_ and *D*_IVA_, coefficients for the effect of IVA and the dummy variable, respectively, encoding the absence (*D*_IVA_ = 0) or presence (*D*_IVA_ = 1) of IVA; *B*_VNS_, coefficient for the effect of VNS; *B*_VNS×TQ_, coefficient for the interaction between VNS and TQ; *B*_VNS×IVA_, coefficient for the interaction between VNS and IVA; *N*_VNS_, VNS rate (10, 20, or 40); *D*_1_–*D*_7_, dummy variables encoding eight rats [for rats 1–7, *D_k_* = 1 when *k* is equal to the rat number and *D_k_* = 0 otherwise. For rat 8, *D*_1_–*D*_7_ are all *D*_k_ = −1, according to the effects coding design (Glantz & Slinker, 2001)]; and *B*_1_–*B*_7_, coefficients to adjust interindividual variations in the repeated‐measures experimental design. The TQ + IVA condition was represented by the dummy variables of *D*_TQ_ = 1 and *D*_IVA_ = 1. As we did not perform a trial examining the effect of IVA alone in this study, *B*_IVA_ and *B*_VNS×IVA_ determined using Equation 4 were interpreted as the effects of IVA in the presence of TQ.

To demonstrate how IVA alone affects the HF gain ratio, we reanalyzed data obtained in our previous study (Kawada, Yamamoto, et al., 2019) using multiple regression analysis (Equation 5) without including the term for TQ as follows:

(5) $$\begin{array}{cc} p & =C+B_{\mathrm{IVA}}\times D_{\mathrm{IVA}}+(B_{\mathrm{VNS}}+B_{\mathrm{VNS}\times \mathrm{IVA}}\times D_{\mathrm{IVA}})\times N_{\mathrm{VNS}}\\ & \;+B_{1}\times D_{1}+B_{2}\times D_{2}+\cdots +B_{6}\times D_{6} \end{array}$$

The number of dummy variables describing interindividual variations was reduced to 6 (*D*_1_–*D*_6_) because only seven rats were examined in that study (Kawada, Yamamoto, et al., 2019).

## 3 RESULTS

**FIGURE 1:**
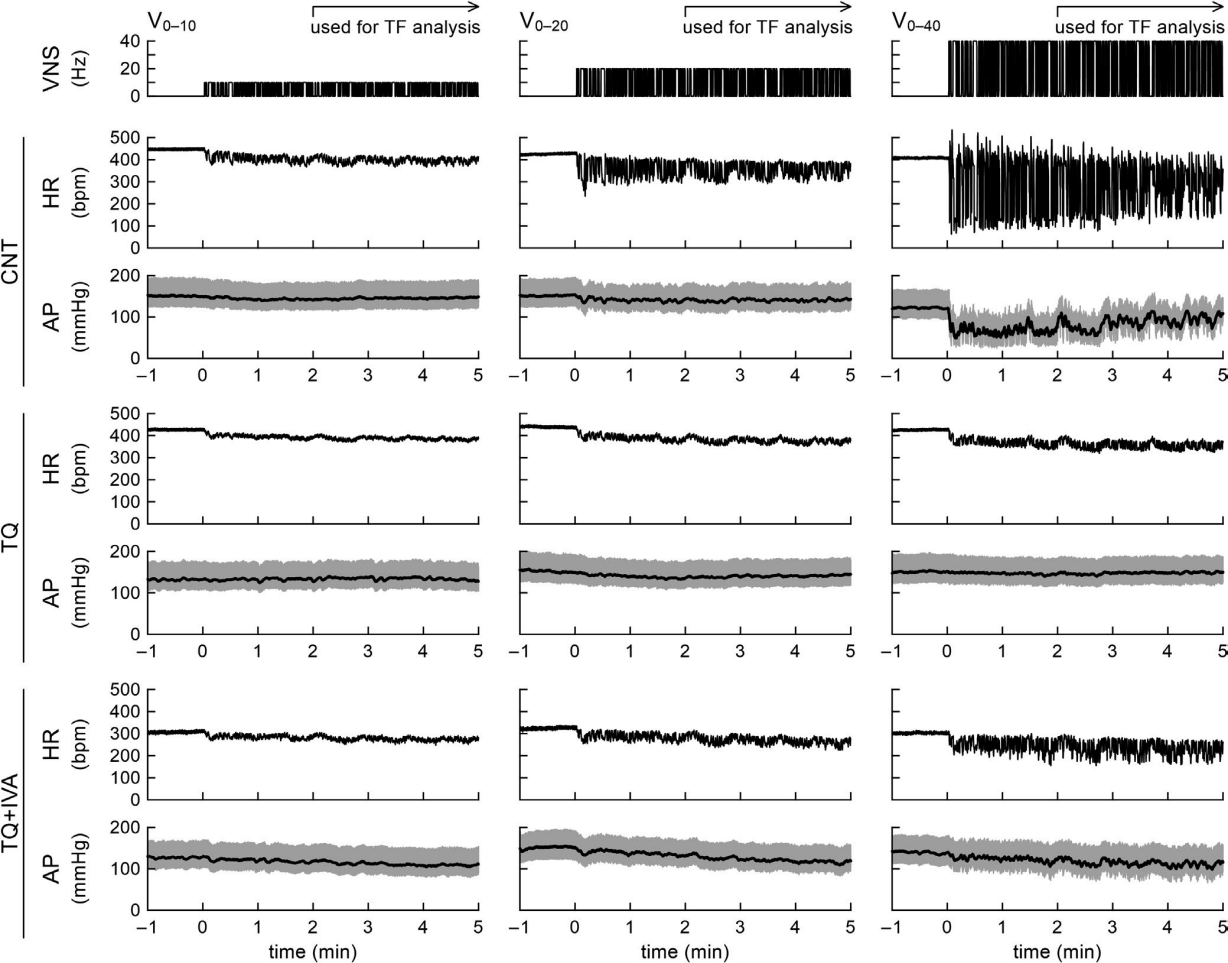
Typical time series of vagal nerve stimulation (VNS), heart rate (HR), and arterial pressure (AP) under conditions of control (CNT), blockade of muscarinic potassium channels by tertiapin‐Q (TQ), and TQ plus ivabradine (TQ + IVA) in one rat. The VNS trials were performed with binary white noise signals between 0–10 Hz (V_0–10_), 0–20 Hz (V_0–20_), and 0–40 Hz (V_0–40_). The VNS commands were identical among the CNT, TQ, and TQ + IVA conditions. For the VNS command and HR plots, 10‐Hz resampled signals are depicted. For the AP plots, the gray and black lines indicate 100‐Hz resampled and 2‐Hz moving average signals, respectively. For the transfer function (TF) analysis, the data were processed from 2 min after the initiation of the VNS. bpm: beats/min

Figure 1 illustrates typical time series obtained from one rat. The top row represents binary VNS command signals, which were identical under CNT, TQ, and TQ + IVA conditions. Under the CNT condition, the magnitude of the dynamic HR response increased with increasing the VNS rate. The HR during V_0–40_ showed rebound increases above the prestimulation HR at intermittent cessations of the binary VNS. The AP did not change perceivably during V_0–10_. The AP showed small fluctuations during V_0–20_ and decreased with large variations during V_0–40_. Under the TQ condition, the dynamic HR response was attenuated at each corresponding VNS rate compared to controls. The AP variation during V_0–40_ became inconspicuous. Under the TQ + IVA condition, the prestimulation HR decreased by approximately 100 bpm in this animal. At each corresponding VNS rate, the dynamic HR response under the TQ + IVA condition was greater than that under the TQ condition but remained attenuated compared to controls. The AP variation during V_0–40_ increased under the TQ + IVA condition compared to that under the TQ condition.

**FIGURE 2:**
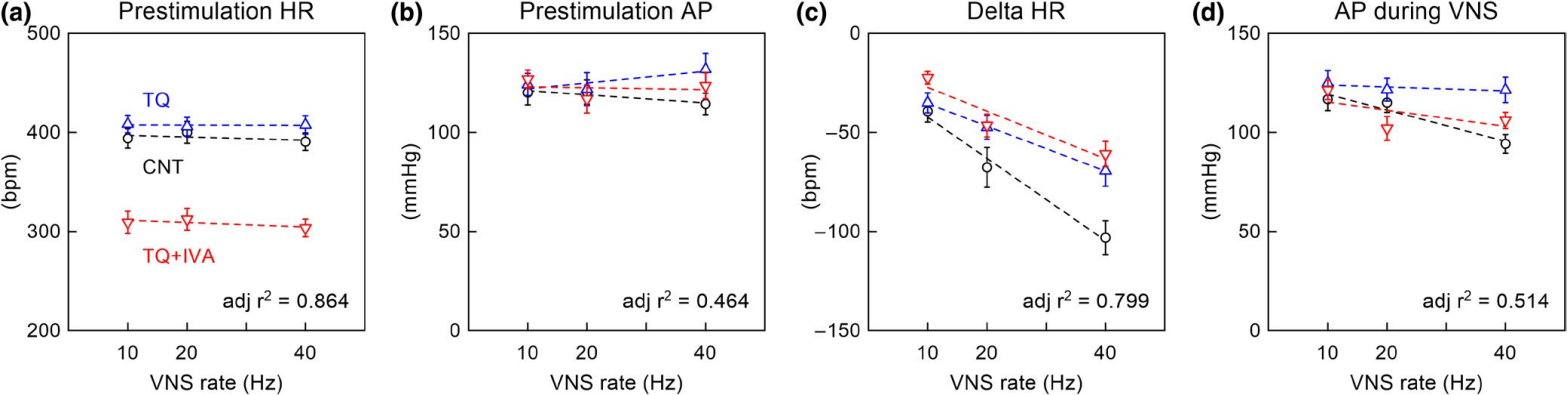
Results of multiple regression analysis on prestimulation heart rate (HR) (a), prestimulation arterial pressure (AP) (b), delta HR (c), and AP during vagal nerve stimulation (VNS) (d). Delta HR was calculated as the difference between HR during VNS and prestimulation. CNT: control (circles); TQ: tertiapin‐Q (up‐pointing triangles); TQ +IVA: TQ plus ivabradine (down‐pointing triangles). Data points represent mean ± SE values (*n* = 8 rats). The dotted lines were drawn based on Equation 4 using the estimated coefficients for each parameter (Table 1). CNT data points: *D*_TQ_ = 0 and *D*_IVA_ = 0 (the black dotted lines); TQ data points: *D*_TQ_ = 1 and *D*_IVA_ = 0 (the blue dotted lines); TQ + IVA data points: *D*_TQ_ = 1 and *D*_IVA_ = 1 (the red dotted lines). adj *r* ^2^: adjusted *r* ^2^ value of multiple regressions; bpm: beats/min

The prestimulation HR did not differ significantly across the three VNS rates (*B*_VNS_ was not significantly different from zero) (Figure 2a and Table 1). TQ did not significantly affect the prestimulation HR (*B*_TQ_ was not significantly different from zero). However, IVA significantly reduced the prestimulation HR by approximately 94 bpm in the presence of TQ (*B*_IVA_ was negative and significantly different from zero). The prestimulation AP did not differ significantly across the three VNS rats (Figure 2b and Table 1). TQ did not significantly affect the prestimulation AP. Despite the marked reduction in prestimulation HR, IVA did not significantly affect the prestimulation AP. The low adjusted *r* ^2^ value indicates that the VNS rate, TQ, and IVA were not major sources of the variation observed in the prestimulation AP.

**TABLE 1:** Results of multiple regression analyses on AP and HR

	*C*	*B*_TQ_	*B*_IVA_	*B*_VNS_	*B*_VNS×TQ_	*B*_VNS×IVA_	adj *r* ^2^
Prestimulation HR, bpm	398.6 ± 8.2	8.9 ± 11.6	−94.1 ± 11.6***	−0.162 ± 0.311	0.148 ± 0.440	−0.209 ± 0.440	0.864
Prestimulation AP, mmHg	123.1 ± 5.7	−4.0 ± 8.1	4.4 ± 8.1	−0.207 ± 0.217	0.499 ± 0.307	−0.340 ± 0.307	0.464
Delta HR, bpm	−21.6 ± 5.6	−2.6 ± 7.9	8.8 ± 7.9	−2.077 ± 0.212***	0.941 ± 0.300**	−0.065 ± 0.300	0.799
AP during VNS, mmHg	127.1 ± 5.3	−2.2 ± 7.6	−5.5 ± 7.6	−0.791 ± 0.202***	0.692 ± 0.286*	−0.311 ± 0.286	0.514

Note

Data are mean ± SE values of multiple regression on 72 data points (3 VNS rates ×3 conditions ×8 rats). The SE values of *B*_TQ_ and *B*_IVA_ are mathematically the same because the inverse of the covariance matrix had same values of the corresponding diagonal elements. The SE values of *B*_VNS×TQ_ and *B*_VNS×IVA_ are also the same.

Abbreviations: adj *r* ^2^, adjusted *r* ^2^ of the multiple regression; AP, arterial pressure; *B*_IVA_, coefficient of the effect of ivabradine (IVA) in the presence of TQ; *B*_TQ_, coefficient of the effect of tertiapin‐Q (TQ); *B*_VNS_, coefficient of the effect of vagal nerve stimulation (VNS) rate; *B*_VNS×IVA_, coefficient of the interaction effect between VNS rate and IVA in the presence of TQ; *B*_VNS×TQ_, coefficient of the interaction effect between VNS rate and TQ; *C*, constant describing the intercept of the multiple regression; HR, heart rate.

*p < 0.05, **p < 0.01, and ***p < 0.001 indicate a significant difference from zero.

Under the CNT condition, the delta HR had a negative slope versus the VNS rate (Figure 2c and Table 1). TQ attenuated the negative slope of the delta HR (*B*_VNS×TQ_ was positive and significantly different from zero), whereas IVA did not affect the slope of the delta HR in the presence of TQ (*B*_VNS×IVA_ was not significantly different from zero). The AP during VNS (hereafter referred to as the “stimulation AP”) demonstrated a negative slope versus the VNS rate (Figure 2d and Table 1). TQ nearly canceled the negative slope of the stimulation AP (*B*_VNS×TQ_ was positive, with a magnitude comparable to the absolute value of *B*_VNS_), whereas IVA did not affect the stimulation AP in the presence of TQ. The adjusted *r* ^2^ value was not high for the stimulation AP.

**FIGURE 3:**
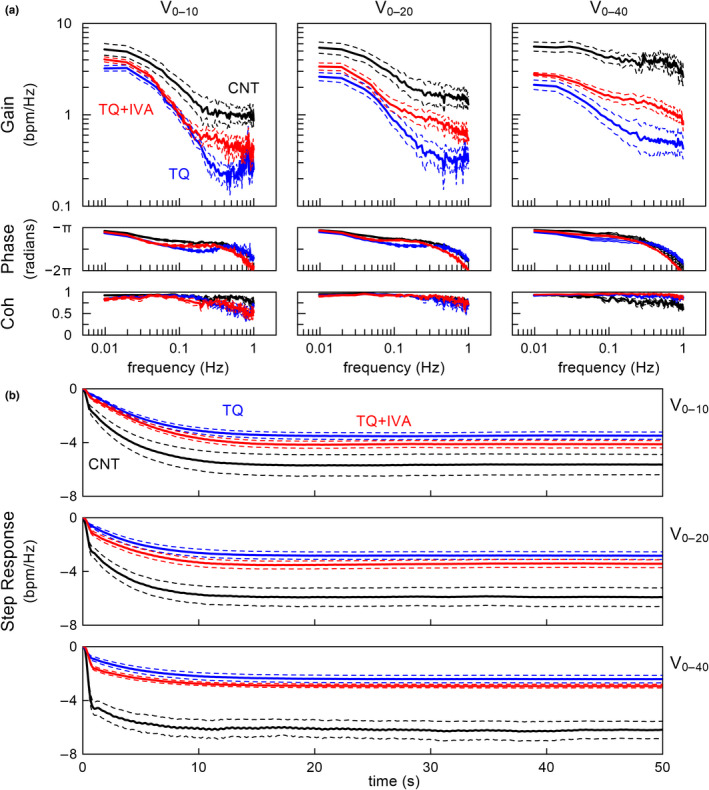
(a) Group‐averaged transfer functions from vagal nerve stimulation (VNS) to heart rate (HR) and the corresponding coherence functions estimated under conditions of control (CNT), tertiapin‐Q (TQ), and TQ plus ivabradine (TQ + IVA). The VNS trials were performed with binary white noise signals between 0–10 Hz (V_0–10_), 0–20 Hz (V_0–20_), and 0–40 Hz (V_0–40_). Coh: coherence. (b) Group‐averaged step responses calculated from the transfer functions. The step response represents the HR response to a unit increase in the VNS. The solid and dashed lines indicate the means ± SE values (*n* = 8 rats). bpm: beats/min

Figure 3a depicts the group‐averaged transfer functions from VNS to HR. The dynamic gain during V_0–10_ decreased as the input modulation frequency increased from approximately 0.02 to 0.2 Hz under the CNT condition. A quasi‐flat zone existed in the dynamic gain above approximately 0.2 Hz. Increasing the VNS rate increased the HF gain at the quasi‐flat zone, resulting in a flatter appearance of the gain plot under the CNT condition. For all VNS rates, TQ attenuated the dynamic gain, and this attenuation was greater in the HF range than in the LF range. TQ + IVA partly recovered the dynamic gain in the HF range.

The phase of the transfer function approached −π radians at the lowest frequency (0.01 Hz), which reflects a negative HR response to VNS. The phase delayed as the input modulation frequency increased. For all VNS rates, TQ increased the phase delay compared to controls in the frequency range from approximately 0.02 to 0.3 Hz. TQ + IVA increased the phase delay compared with the CNT and TQ conditions above approximately 0.5 Hz.

The coherence during V_0–10_ was near unity in the frequency range below 0.1 Hz and slightly decreased from 0.1 to 1 Hz under the CNT condition. TQ and TQ + IVA decreased the coherence in the frequency range above approximately 0.3 Hz compared to controls. The coherence during V_0–20_ did not differ among the CNT, TQ, and TQ + IVA conditions. The coherence during V_0–40_ was near unity in the frequency range below 0.1 Hz and decreased to approximately 0.7 in the frequency range from 0.1 to 1 Hz under the CNT condition. TQ and TQ + IVA elevated the coherence in the frequency range above approximately 0.1 Hz compared to controls.

Figure 3b illustrates the group‐averaged step responses of the HR calculated from the transfer functions. The step responses under the CNT condition showed an initial drop within 1 s, followed by a gradual reduction to reach the steady‐state response at approximately 10 s. The initial drop was more exaggerated as the VNS rate increased. For all VNS rates, TQ obscured the initial drop without significantly attenuating the gradual reduction of HR. TQ + IVA slightly recovered the initial drop compared with the TQ condition.

**FIGURE 4:**
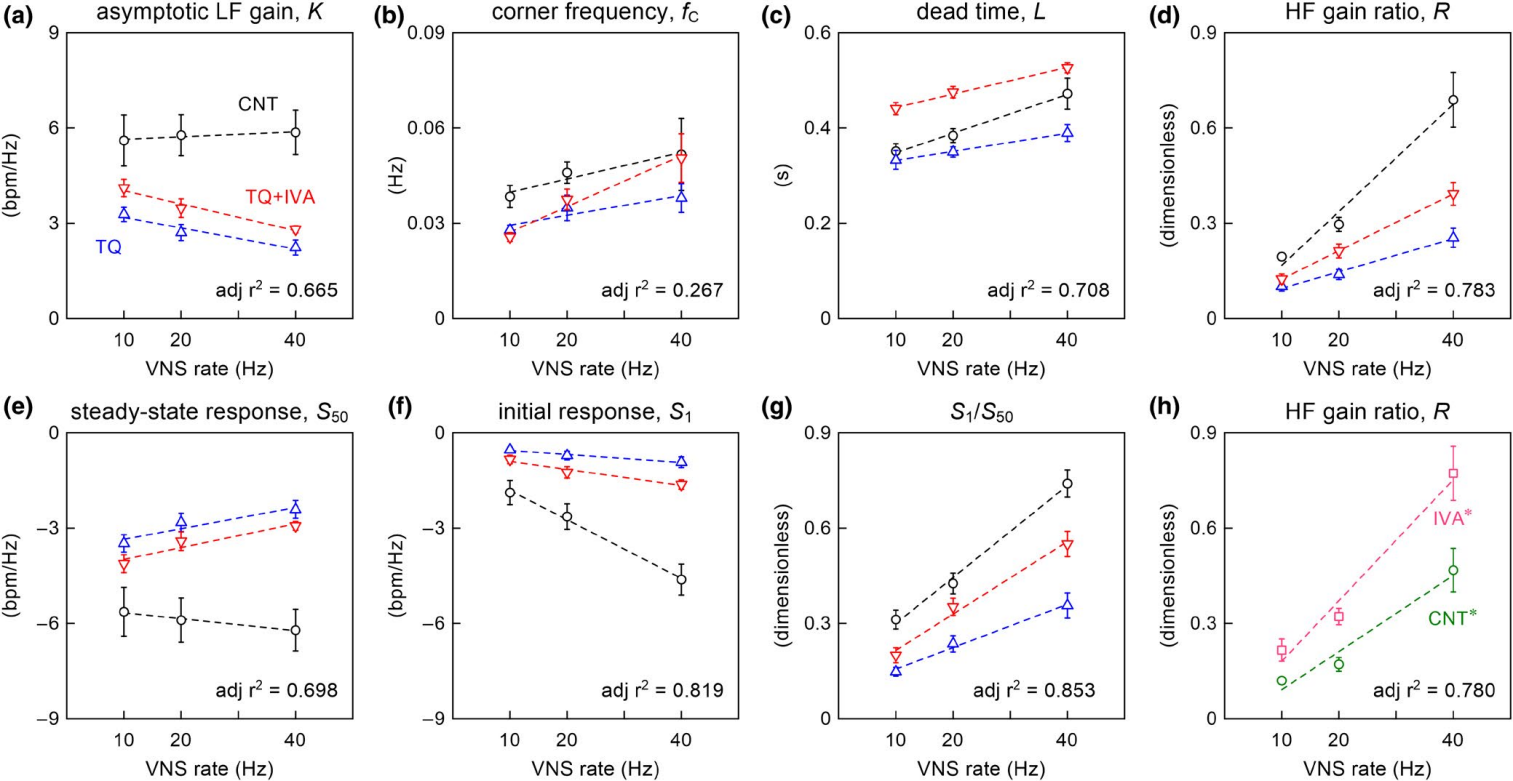
Results of multiple regression analysis on transfer function parameters (a–d) and step response parameters (e–g). CNT: control (circles); TQ: tertiapin‐Q (up‐pointing triangles); TQ +IVA: TQ plus ivabradine (down‐pointing triangles); VNS: vagal nerve stimulation; *K*: asymptotic low frequency (LF) gain; *f*_C_: corner frequency; *L*: dead time; *R*: high frequency (HF) gain ratio; *S*_50_: steady‐state response; *S*_1_: initial response at 1 s; *S*_1_/*S*_50_: ratio of *S*_1_ to *S*_50_. Data points represent mean ± SE values (*n* = 8 rats). The dotted lines were drawn based on Equation 4 using the estimated coefficients for each parameter (Table 2). CNT data points: *D*_TQ_ = 0 and *D*_IVA_ = 0 (the black dotted lines); TQ data points: *D*_TQ_ = 1 and *D*_IVA_ = 0 (the blue dotted lines); TQ +IVA data points: *D*_TQ_ = 1 and *D*_IVA_ = 1 (the red dotted lines). adj *r* ^2^: adjusted *r* ^2^ value of multiple regression; bpm: beats/min. In panel (h), the effect of IVA alone on the HF gain ratio is shown based on previously obtained data (Kawada, Yamamoto, et al., 2019). CNT* (circles) and IVA* (squares) represent the data obtained under the conditions of control and IVA alone (*n* = 7 rats). The dotted lines were drawn based on Equation 5 using the estimated coefficients for each parameter (Table 3)

Figure 4a–d summarizes the results of the multiple regression analyses on the transfer function parameters. The asymptotic LF gain did not differ significantly across the three VNS rates under the CNT condition (Figure 4a and Table 2). The negative *B*_TQ_ indicates that TQ significantly lowered the asymptotic LF gain. IVA did not affect the asymptotic LF gain in the presence of TQ. The effect on the corner frequency was not significant for the VNS rate, TQ, or IVA, with a low adjusted *r* ^2^ value (Figure 4b and Table 2). The dead time positively correlated with the VNS rate (Figure 4c and Table 2). The negative *B*_VNS×TQ_ indicates that TQ significantly reduced the slope of the dead time versus the VNS rate. *B*_IVA_ was positive, whereas *B*_VNS×IVA_ was not significantly different from zero, indicating that IVA prolonged the dead time in the presence of TQ without a significant effect on the slope of the dead time. The HF gain ratio positively correlated with the VNS rate (Figure 4d and Table 2). TQ significantly reduced the slope of the HF gain ratio versus the VNS rate. IVA did not significantly affect the slope of the HF gain ratio in the presence of TQ. The HF gain ratio at each VNS rate was lower under the TQ + IVA condition compared to controls.

**TABLE 2:** Results of multiple regression analyses on transfer function and step response parameters

	*C*	*B*_TQ_	*B*_IVA_	*B*_VNS_	*B*_VNS×TQ_	*B*_VNS×IVA_	adj *r* ^2^
*K*, bpm/Hz	5.56 ± 0.46	−2.05 ± 0.65**	0.93 ± 0.65	0.008 ± 0.017	−0.041 ± 0.024	−0.009 ± 0.024	0.665
*f*_C_, (×0.01) Hz	3.56 ± 0.62	−0.93 ± 0.88	−0.71 ± 0.88	0.042 ± 0.024	−0.011 ± 0.033	0.049 ± 0.033	0.267
*L*, (×0.1) s	3.07 ± 0.19	0.06 ± 0.26	1.02 ± 0.26 ***	0.041 ± 0.007***	−0.022 ± 0.010*	0.009 ± 0.010	0.708
*R* (×0.01)	−0.12 ± 3.99	4.45 ± 5.65	−0.86 ± 5.65	1.691 ± 0.151***	−1.170 ± 0.214***	0.374 ± 0.214	0.783
*S*_50_, bpm/Hz	−5.48 ± 0.44	1.79 ± 0.62**	−0.67 ± 0.62	−0.019 ± 0.017	0.052 ± 0.024*	0.004 ± 0.024	0.698
*S*_1_, (×0.1) bpm/Hz	−8.88 ± 2.62	4.59 ± 3.71	−2.17 ± 3.71	−0.924 ± 0.099***	0.798 ± 0.140***	−0.127 ± 0.140	0.819
*S*_1_/*S*_50_, (×0.01)	15.49 ± 3.23	−6.70 ± 4.57	1.28 ± 4.57	1.448 ± 0.122***	−0.768 ± 0.173***	0.462 ± 0.173*	0.853

Note

Data are mean ± SE values of multiple regression on 72 data points (3 VNS rates ×3 conditions ×8 rats). The SE values of *B*_TQ_ and *B*_IVA_ are mathematically the same. The SE values of *B*_VNS×TQ_ and *B*_VNS×IVA_ are also the same.

Abbreviations: adj *r* ^2^, adjusted *r* ^2^ of the multiple regression; *B*_IVA_, coefficient of the effect of ivabradine (IVA) in the presence of TQ; *B*_TQ_, coefficient of the effect of tertiapin‐Q (TQ); *B*_VNS_, coefficient of the effect of vagal nerve stimulation (VNS) rates; *B*_VNS×IVA_, coefficient of the interaction between VNS rates and IVA in the presence of TQ; *B*_VNS×TQ_, coefficient of the interaction between VNS rates and TQ; *C*, constant describing the intercept of the multiple regression; *f*_C_, corner frequency; *K*, asymptotic low frequency gain; *L*, dead time; *R*, fraction of high frequency gain relative to *K*; *S*_1_, initial response; *S*_50_, steady‐state response.

* *p* < 0.05, **p < 0.01, and ***p < 0.001 indicate a significant difference from zero.

Figure 4e–g summarizes the results of the multiple regression analyses on step response parameters. VNS rate had no significant effect on the steady‐state response under the CNT condition (Figure 4e and Table 2). For all VNS rates, TQ significantly attenuated the magnitude of the steady‐state response. IVA did not affect the steady‐state response in the presence of TQ. The initial response was enhanced as the VNS rate increased under the CNT condition (Figure 4f and Table 2). *B*_VNS×TQ_ was positive with a magnitude similar to the absolute value of *B*_VNS_, indicating that TQ nearly abolished the effect of the VNS rate on the initial response. IVA did not significantly affect the slope of the initial response in the presence of TQ. The ratio of *S*_1_ to *S*_50_ positively correlated with the VNS rate under the CNT condition (Figure 4g and Table 2). TQ reduced the slope of the ratio of *S*_1_ to *S*_50_ versus the VNS rate, whereas IVA partly recovered this slope in the presence of TQ. However, the ratio of *S*_1_ to *S*_50_ at each VNS rate remained lower under the TQ + IVA condition compared to controls.

Figure 4h depicts the effect of IVA alone on the HF gain ratio derived from previously obtained data (Kawada, Yamamoto, et al., 2019). *B*_VNS_ was positive and significantly different from zero (Table 3), indicating that the HF gain ratio increased with the VNS rate. Further, *B*_VNS×IVA_ was positive and significantly different from zero, indicating that IVA alone significantly increased the slope of the HF gain ratio versus the VNS rate.

**TABLE 3:** Results of multiple regression analyses regarding the effect of ivabradine on the high frequency gain ratio of the transfer function

	*C*	*B*_IVA_	*B*_VNS_	*B*_VNS×IVA_	adj *r* ^2^
*R* (×0.01)	−2.85 ± 5.55	1.90 ± 7.86	1.206 ± 0.210**	0.706 ± 0.270*	0.780

Data are mean ± SE values of multiple regression on 42 data points (3 VNS rates ×2 conditions ×7 rats) derived from our previous study (Kawada, Yamamoto, et al., 2019).

Abbreviations: adj *r* ^2^, adjusted *r* ^2^ of the multiple regression; *B*_IVA_, coefficient of the effect of ivabradine (IVA); *B*_VNS_, coefficient of the effect of vagal nerve stimulation (VNS) rates; *B*_VNS×IVA_, coefficient of the interaction between VNS rates and IVA; *C*, constant describing the intercept of the multiple regression; *R*, fraction of high frequency gain relative to asymptotic low frequency gain.

* *p* < 0.05 and **p < 0.001 indicate a significant difference from zero.

## 4 DISCUSSION

The effect of IVA on the dynamic vagal control of HR was characterized by an increase in the HF gain ratio of the transfer function from VNS to HR, as described in our previous studies (Kawada, Yamamoto, et al., 2019; Kawada et al., 2021) and demonstrated in Figure 4h. In the present study, TQ decreased the HF gain ratio compared to controls (Figure 4d), suggesting the involvement of *I*_K,ACh_ in the determination of the HF gain ratio. Furthermore, IVA in the presence of TQ did not increase the HF gain ratio compared to controls.

### 4.1 Effects of *I*_K,ACh_ blockade on the dynamic vagal control of HR

The *I*_K,ACh_ pathway has two distinct features in controlling HR compared to the cAMP‐mediated *I*_f_ pathway. First, the activation of *I*_K,ACh_ requires a higher concentration of acetylcholine compared to the inhibition of *I*_f_ (DiFrancesco et al., 1989). Because the myocardial interstitial acetylcholine concentration in the atria near the sinoatrial node positively correlated with the VNS rate (Shimizu et al., 2009), the extent of *I*_K,ACh_ activation might have also increased alongside the VNS rate. Second, because it does not require cytoplasmic components, the *I*_K,ACh_ pathway contributes to the fast HR response to VNS in contrast to the *I*_f_ pathway. The fast HR response is associated with dynamic gain in the HF range. The flattening of the gain plot with increased VNS rate under the CNT condition (Figure 3a, black lines) indicates that *I*_K,ACh_ has an increasing contribution to the dynamic HR response with an increase in the VNS rate.

TQ attenuated the dynamic gain of the transfer function from VNS to HR, with greater attenuation in the HF range (Figure 3a, blue lines). A previous study in rabbits demonstrated similar results, except that the transfer function from VNS to HR in rabbits did not reveal a quasi‐flat zone in the HF range (Mizuno et al., 2007). Furthermore, only one VNS rate (10 Hz) was tested in the rabbit experiment, whereas this study examined three VNS rates. The fact that TQ significantly decreased the slope of the HF gain ratio versus the VNS rate (Figure 4d) further supports the notion that *I*_K,ACh_ has an increasing contribution to the dynamic vagal control of HR with increasing VNS rate.

### 4.2 Effects of IVA on the dynamic vagal control of HR under *I*_K,ACh_ blockade

Overexpression of HCN4 in mice attenuated the bradycardic response to VNS in the presence of β‐adrenergic stimulation (Kozasa et al., 2018). Conversely, HCN4 knockout enhanced the bradycardic response to VNS (Kozasa et al., 2018). These results suggest that the activation of *I*_f_ counteracts *I*_K,ACh_. However, the effects of HCN channel blockade on the frequency‐dependent nature of the vagal control of HR are yet to be elucidated. We have previously shown that IVA increased the HF gain ratio of the transfer function from VNS to HR (Figure 4h; Kawada, Yamamoto, et al., 2019; Kawada et al., 2021). On the other hand, in the present study, IVA in the presence of TQ was unable to increase the HF gain ratio compared to controls (Figure 4d). The ratio of *S*_1_ to *S*_50_ was also lower under the TQ + IVA condition compared to controls, although IVA partly recovered this ratio (Figure 4g). Hence, the IVA‐induced increase in the HF gain ratio or the enhancement of the initial step response observed in our previous studies most likely depended on the relative activation of *I*_K,ACh_.

In a previous study, physiological levels of VNS slow the firing rate of rabbit pacemaker cells without a detectable hyperpolarizing effect on the MDP (Shibata et al., 1985), suggesting that *I*_K,ACh_ does not significantly contribute to the HR response unless a much higher VNS is applied (Demir et al., 1999). By contrast, Han and Bolter (Han & Bolter, 2011) demonstrated that *I*_K,ACh_ always participates in the vagal slowing of guinea‐pig atria. In their study, MDP changed by 0.076 mV for each 1% VNS‐induced bradycardia. The inhibition of HCN channels by IVA may untether the possible hyperpolarizing effect of *I*_K,ACh_ and may enhance the dynamic HR response to VNS mediated by *I*_K,ACh_. In the present study, after *I*_K,ACh_ blockade, IVA no longer increased the HF gain ratio compared to controls.

Using an isolated, vagal‐innervated preparation of guinea‐pig atria, Han and Bolter (Han & Bolter, 2015) demonstrated that blocking *I*_K,ACh_ and *I*_f_ by TQ and ZD7288, respectively, caused a 90% reduction of the atrial rate response to 10‐s trains of VNS. They concluded that *I*_K,ACh_ and *I*_f_ modulation sufficiently accounts for all vagal slowing observed in their preparation. In their study, TQ alone reduced the atrial rate response to VNS by approximately 50%, whereas ZD7288 alone had no effect. In the present study, TQ alone significantly reduced both the delta HR and the asymptotic LF gain (Figures 2c and 4a). However, IVA did not show an additional reduction of the HR response to VNS in the presence of TQ. While the prestimulation HR reduced by approximately 94 bpm by IVA (Table 1), the blockade of *I*_f_ might have been incomplete in the present study carried out in vivo. The differences in species (rats versus guinea pigs), drugs (IVA versus ZD7288), and modes of VNS could have also accounted for the discrepancy.

### 4.3 Possible implication

The HF component of HR variability (HRV) has been used as an index of vagal outflow from the central nervous system. However, modulations of ion channel mechanisms in the sinoatrial nodal cells can also affect HRV through changes in the dynamic gain of the vagal transfer function in the HF range. The HF gain ratio is positively correlated with the VNS rate in normal control rats, as evidenced by the CNT data in Figure 4d. However, the correlation is lost in rats with chronic heart failure after myocardial infarction (Kawada et al., 2013). In failing human hearts, atrial myocytes exhibited a less negative resting membrane potential with *I*_K,ACh_ dysfunction (Koumi et al., 1994). Hence, the *I*_K,ACh_ dysfunction, alongside the vagal withdrawal, may contribute to the manifestation of decreased HF components of HRV observed in chronic heart failure.

In patients with systolic heart failure, treatment with IVA for 8 months significantly lowers HR and improves HRV (Böhm et al., 2015). The improved HRV could be attributed to the general improvement of the autonomic balance. In addition, there is a possibility that untethering of the hyperpolarizing effect of *I*_K,ACh_ by IVA contributed as a peripheral mechanism for HRV modulation, though it remains hypothetical until proven by chronic animal experiments. Changes in HRV due to the peripheral mechanism are also discussed in association with systemic inflammation (Gholami et al., 2012; Hajiasgharzadeh et al., 2011). Currently, it is still unknown whether an increase in HRV via the peripheral mechanism, without an actual increase in vagal outflow, exerts any cardioprotective effect. Generating artificial respiratory sinus arrhythmia (RSA) with patterned VNS has been shown to benefit the pulmonary gas exchange compared with no RSA with constant VNS (Hayano et al., 1996). Hence, it is possible that the increased HRV in the HF range via the peripheral mechanism serves as an active physiological role in improving the general condition.

### 4.4 Limitations

First, we focused on the effect of IVA on the vagal control of HR. The treatment effect of IVA may include a pleiotropic effect, such as the reduction of myocardial infarct size in the absence of bradycardia (Heusch & Kleinbongard, 2016; Heusch et al., 2008; Kleinbongard et al., 2015). Thus, the effect of IVA beyond HR reduction needs to be considered to fully understand the benefits of IVA in heart failure patients (Swedberg et al., 2010; Tsutsui et al., 2019). Second, it remains unknown whether IVA chronically affects the dynamic vagal control of HR because our experiment was performed acutely under anesthesia. Third, the stimulation pattern of VNS was completely different from that of physiological vagal nerve activity. Hence, our results could not be directly extrapolated to the physiological vagal control of HR. Nevertheless, the results of the vagal transfer function in the HF range may be pertinent to interpret RSA, which is driven by the cardiac vagal nerve activity. Fourth, we focused on *I*_K,ACh_ and *I*_f_ in the vagal control of HR, but other currents such as L‐type (*I*_CaL_) and T‐type (*I*_CaT_) calcium currents are involved in producing the pacemaker potential (Murphy & Lazzara, 2016). These calcium channels are activated by a rise in the membrane potential late during the slow diastolic depolarization. Their activation threshold was around −40 mV: more negative than −40 mV for *I*_CaT_ and more positive than −40 mV for *I*_CaL_ (Murphy & Lazzara, 2016). By contrast, *I*_f_ channels are typically activated by hyperpolarization at membrane potentials below −60 mV. Hence, the interaction of *I*_f_ with *I*_CaT_ and *I*_CaL_ might not be as large as the interaction of *I*_f_ with *I*_K,ACh_. Finally, TQ is not a selective inhibitor of *I*_K,ACh_ and can block other types of potassium channels such as calcium‐activated large conductance potassium channels, also designated as “Big K” (BK) channels (Kanjhan et al., 2005). BK channels are expressed in vascular smooth muscles and neuronal cells and generally suppress membrane excitability. The blockade of BK channels by iberiotoxin facilitates myocardial interstitial acetylcholine release and augments the HR reduction during VNS (Kawada et al., 2010). In sinus nodal cells, the blockade of BK channels by iberiotoxin prolongs the slow diastolic depolarization and decreases the firing rate (Lai et al., 2014). However, TQ alone did not significantly affect the prestimulation HR in this study (Figure 2a). Further, the net effect of TQ was the attenuation of the delta HR during VNS (Figure 2c). Hence, the BK channel blocking ability of TQ might not have significantly affected the present results.

In conclusion, this study examined the frequency‐dependent effect of IVA on the dynamic vagal control of HR under *I*_K,ACh_ blockade by TQ. IVA did not increase the HF gain ratio, suggesting that the involvement of *I*_K,ACh_ is a prerequisite for the previously observed IVA‐induced increase in the HF gain ratio of the transfer function from VNS to HR. These findings reinforce the hypothesis that untethering the hyperpolarizing effect of *I*_K,ACh_ after IVA may be a mechanism for the frequency‐dependent effect of IVA on the dynamic vagal control of HR.

## CONFLICT OF INTEREST

The authors declare no conflict of interest.

## AUTHOR CONTRIBUTIONS

T.K. conceived and designed research, performed experiments, and analyzed data; T.K., H.Y., T.M., Y.H., M.L., C.Z., K.U., M.S., and K.S. interpreted resuls of experiments; T.K. prepared figures and drafted the manuscript; T.K. edited and revised the manuscript; T.K., H.Y., T.M., Y.H., M.L., C.Z., K.U., M.S., and K.S. approved the final version of the manuscript.
